# Corynebacterium bovis Keratitis Mimicking Acanthamoeba Infection Following Ocular Trauma

**DOI:** 10.7759/cureus.112238

**Published:** 2026-07-07

**Authors:** Reyleen Loreto, Sarah Mourtada, Ali Hussaini, Devin Perez, Don J Perez ll, Parul Aneja

**Affiliations:** 1 Internal Medicine, St. Joseph Hospital/Baycare Health System, Tampa, USA; 2 School of Dentistry, Marquette University, Milwaukee, USA; 3 Infectious Disease, St. Joseph Hospital/Baycare Health System, Tampa, USA

**Keywords:** corneal ulcer, corynebacterium bovis, diabetes mellitus, diphtheroids, keratitis, ocular trauma

## Abstract

Corneal isolates of *Corynebacterium* species are frequently dismissed as contaminants, yet they are capable of causing severe, vision-threatening keratitis. A 45-year-old man with no history of contact lens use developed acute left eye pain and visual loss after a stone fragment struck his eye during work, with immediate contamination by garden hose water. Slit-lamp examination revealed a ring-shaped stromal infiltrate with visual acuity reduced to light perception, a presentation consistent with *Acanthamoeba* keratitis. Empiric therapy with fortified topical vancomycin and ceftazidime, antifungal and anti-amoebic agents, and systemic antibiotics was initiated before culture results were available. Admission evaluation identified previously undiagnosed diabetes mellitus with a hemoglobin A1c level of 12.1%. Corneal cultures grew *Corynebacterium bovis* as the sole organism, confirmed by matrix-assisted laser desorption/ionization time-of-flight (MALDI-TOF) mass spectrometry; *Acanthamoeba* and fungal cultures were negative at four weeks. The patient improved on culture-directed topical vancomycin but required surgical debridement 28 days after discharge for a persistent corneal abscess with hypopyon; intraoperative cultures were sterile, and the globe was preserved. *C. bovis* keratitis can present with morphologic and clinical features that closely mimic *Acanthamoeba* infection; in the setting of ocular trauma and metabolic risk factors, diphtheroid corneal isolates should be considered pathogens until culture and clinical correlation suggest otherwise.

## Introduction

*Corynebacterium *species are pleomorphic, Gram-positive bacilli, commonly referred to as diphtheroids, that colonize the normal skin and conjunctival surface [1,2]. Although generally regarded as commensals, they may cause opportunistic infection when host defenses are compromised or physical barriers disrupted. Predisposing conditions include diabetes mellitus, immunosuppressive therapy, corneal trauma, contact lens use, ocular surgery, and prolonged hospitalization [2]. Because *Corynebacterium *species are frequently recovered from ocular surface cultures, they are often dismissed as contaminants; however, several species have been documented as causative agents of keratitis and other ocular infections [2,3].

*Corynebacterium bovis* (*C. bovis*) is rarely reported as a cause of human ocular disease. Its association with keratitis occurs at a significantly lower frequency than that of other *Corynebacterium *species [1], making clinical recognition particularly challenging. A ring-shaped stromal infiltrate, a circumferential zone of corneal inflammation, is classically associated with *Acanthamoeba *keratitis but is nonspecific and may also accompany severe bacterial or fungal infection, a feature that often complicates early diagnosis [3]. We present a case of trauma-associated *C. bovis* keratitis in which a ring-shaped stromal infiltrate initially mimicked *Acanthamoeba* keratitis, requiring broad empiric therapy before culture confirmed the responsible organism.

## Case presentation

A 45-year-old man employed as a flooring installer, with no significant past medical history and no history of contact lens use, presented to the emergency department with acute left eye pain. While drilling travertine stone flooring, a stone fragment struck his left eye, and he immediately rinsed the eye with water from a garden hose. Initial pain was mild (2/10) with slight blurring of vision, and he was able to complete his workday.

The following day, he developed increased lacrimation, whitish mucoid discharge from the left eye, and a persistent foreign body sensation with photophobia. More than 24 hours after the injury, his symptoms worsened acutely: pain escalated to 9/10, discharge became mucopurulent, and progressive visual loss in the left eye disrupted sleep, prompting emergency department presentation. He denied systemic symptoms.

On admission (Day 0), complete blood count showed no leukocytosis or neutrophilia. Erythrocyte sedimentation rate was within normal limits; C-reactive protein was mildly elevated at 0.82 mg/dL. Serum sodium was 135 mmol/L. Serum glucose was 326 mg/dL with a hemoglobin A1c of 12.1%, indicating previously undiagnosed diabetes mellitus (Table 1). 

**Table 1:** Relevant laboratory findings.

Laboratory Test	Patient value	Reference range
White blood cells	6.5 ×10³/µL	4.5-11.0 ×10³/µL
Hemoglobin	14.6 g/dL	14.0-18.0 g/dL
Platelet count	136 ×10³/µL (low)	140-450 ×10³/µL
Serum glucose	326 mg/dL (high)	70-99 mg/dL
Hemoglobin A1c	12.1% (high)	4.7-5.6%
Blood urea nitrogen	13 mg/dL	6-26 mg/dL
Creatinine	0.90 mg/dL	0.60-1.30 mg/dL
Sodium	135 mmol/L (low)	136-145 mmol/L
Potassium	4.2 mmol/L	3.5-5.1 mmol/L
Chloride	102 mmol/L	98-109 mmol/L
Bicarbonate	25 mmol/L	21-32 mmol/L
Erythrocyte sedimentation rate (ESR)	2 mm/h	0-15 mm/h
C-reactive protein (CRP)	0.82 mg/dL (high)	0.00-0.50 mg//dL

**Figure 1:**
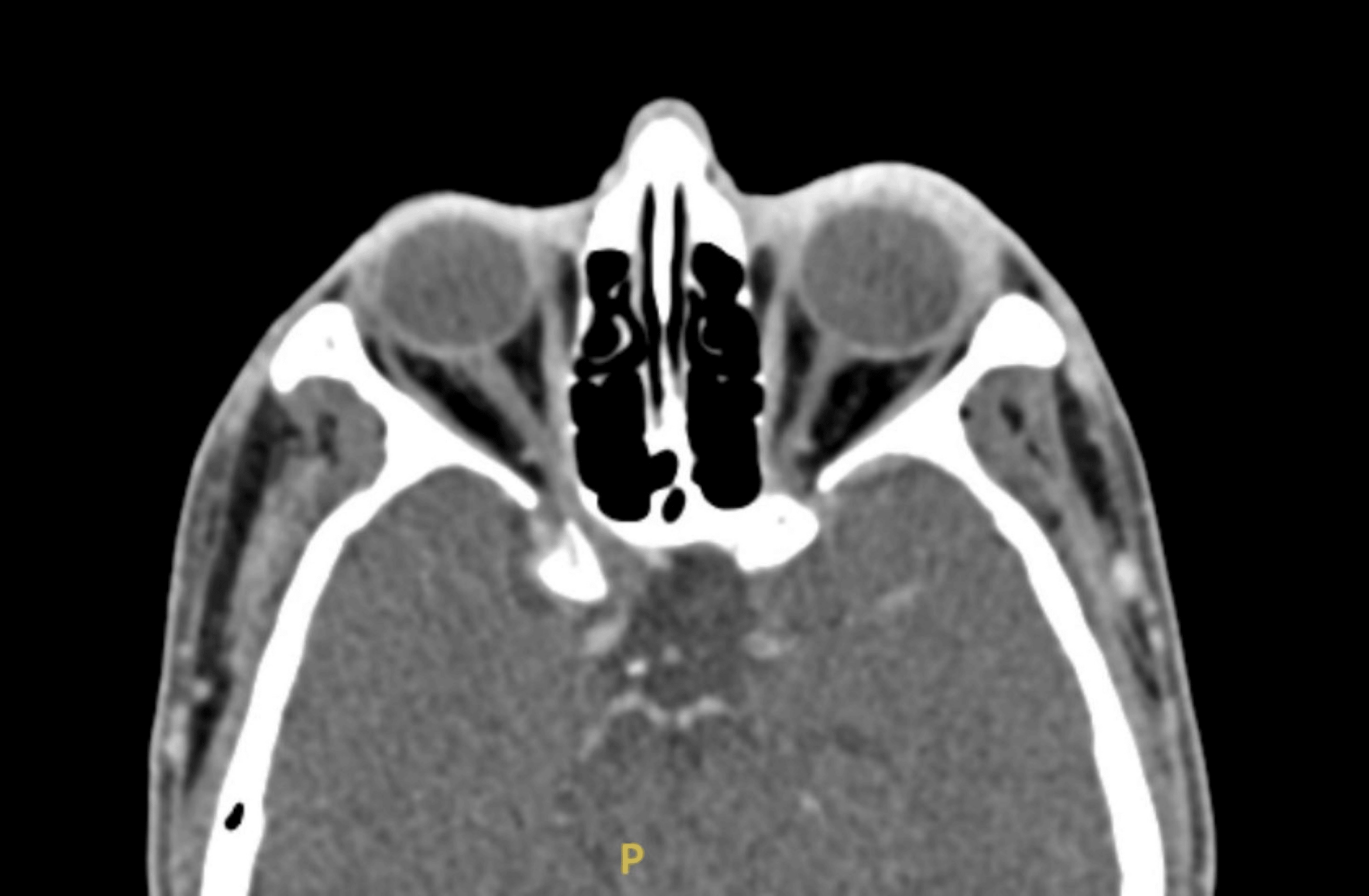
Axial computed tomography of the orbits with intravenous contrast demonstrating no intraocular or orbital foreign body and no evidence of post-septal involvement.

Computed tomography of the orbits with contrast showed no intraocular or orbital foreign bodies (Figure 1). 

Ophthalmology was consulted emergently. Although the initial emergency department evaluation suggested a corneal abrasion, formal ophthalmologic examination revealed a ring-shaped stromal ulcer with mucopurulent discharge. Visual acuity in the left eye was limited to light perception to hand motion. External cultures, including fungal cultures, were obtained from the conjunctival, eyelid, lash, lacrimal, and external lamella surfaces. Corneal scrapings were obtained and submitted for Gram stain, Giemsa stain, and microbiological culture, including culture for *Acanthamoeba*; preliminary smear results were not formally documented in the chart. A complete bedside ocular lavage with 10% povidone-iodine was performed, with transition to 5% ophthalmic povidone-iodine drops four times daily.

Corneal scrapings were inoculated into thioglycollate broth and plated on MacConkey, chocolate, blood, colistin-nalidixic acid, and CDC anaerobic media. *Acanthamoeba* and fungal cultures were submitted concurrently.

Given the severity of the presentation (ring-shaped stromal infiltrate, near-total visual loss, and mucopurulent discharge) and the absence of preliminary microbiologic data, broad empiric topical antimicrobial therapy was initiated without awaiting culture results, consistent with American Academy of Ophthalmology (AAO) Preferred Practice Pattern guidelines for vision-threatening bacterial keratitis [4]. Vancomycin ophthalmic drops (33 mg/mL) were initiated every two hours given the severity of the infection and adjusted to every six hours the following morning as the acute phase stabilized; the agent was selected for Gram-positive coverage, including *Corynebacterium* species, staphylococci, and streptococci, given documented susceptibility and rising fluoroquinolone resistance among *Corynebacterium* isolates [5,6]. Ceftazidime ophthalmic drops (100 mg/mL, every six hours) provided Gram-negative coverage, including *Pseudomonas aeruginosa *[7]. Neomycin/polymyxin B/gramicidin ophthalmic solution (four times daily) broadened antibacterial coverage. Voriconazole ophthalmic drops (1% (10 mg/mL), four times daily) were added empirically because the history of environmental corneal trauma with soil and contaminated water carries a well-established risk for filamentous fungal keratitis [8]. Cyclopentolate 2% ophthalmic solution (twice daily) was administered for cycloplegia to reduce ciliary spasm-related pain and prevent synechiae.

The ring-shaped circumferential stromal infiltrate and recent contaminated water exposure raised *Acanthamoeba* keratitis as the primary differential diagnosis. Standard topical anti-amoebic agents, specifically polyhexamethylene biguanide (PHMB) and chlorhexidine drops, were not immediately available. Bridge antiseptic therapy was therefore initiated using Boston Simplus® solution (containing chlorhexidine and polyaminopropyl biguanide) four times daily while awake and continued for one week; both agents have documented in vitro cysticidal activity against *Acanthamoeba* [9,10]. A request was placed for oral miltefosine 50 mg three times daily as adjunctive systemic anti-amoebic therapy. Miltefosine is an alkylphosphocholine recognized by the Centers for Disease Control and Prevention (CDC) for use in free-living amebic infections, with a proposed mechanism involving disruption of parasite membrane phospholipids and mitochondrial function, though its precise anti-amoebic mechanism remains under investigation [11,12]. With active infection and an unresolved differential diagnosis, miltefosine was initiated off-label as a systemic bridge with plans for de-escalation once microbiologic results were available.

Infectious Disease initiated systemic piperacillin-tazobactam (4.5 g IV every eight hours) and intravenous vancomycin, given concern for deep stromal involvement and risk of intraocular extension; concurrent systemic coverage was considered appropriate given the depth of stromal involvement and potential for scleral spread [4]. Insulin aspart (correction scale with meals and at bedtime) and insulin glargine 20 units twice daily were started to address significant hyperglycemia, as uncontrolled diabetes was considered a contributing factor to infection severity and impaired corneal healing.

On Day 2, oral miltefosine was initiated at 50 mg three times daily and reduced to twice daily after 48 hours. The patient demonstrated improvement in pain from 9/10 to 5/10; visual acuity improved to counting fingers at one foot. The corneal ulcer decreased in size with reduced mucopurulent discharge. The ring-shaped infiltrate showed circumferential improvement: the peripheral cornea was clearing, while the central cornea remained involved.

Growth occurred only on enriched, nonselective media (blood agar and chocolate agar), with no growth on MacConkey or anaerobic plates; this pattern is consistent with a fastidious Gram-positive organism. On Day 4, corneal cultures yielded *C. bovis* as the sole organism, identified to the species level by matrix-assisted laser desorption/ionization time-of-flight mass spectrometry (MALDI-TOF MS), a proteomic method with high accuracy for *Corynebacterium *speciation. Fungal cultures showed no growth at two weeks and were finalized as negative at four weeks; *Acanthamoeba *cultures were also negative. Antimicrobial susceptibility testing was not performed on the *C. bovis *isolate; therapy was therefore culture-directed, informed by published susceptibility data rather than isolate-specific results. The patient demonstrated sustained clinical improvement on the vancomycin-based regimen, and published literature consistently demonstrates near-uniform in vitro susceptibility of *Corynebacterium *species to vancomycin across large isolate series [5,7,13]. Management was guided by organism identification, clinical response, and the AAO Preferred Practice Pattern, which states that antimicrobial modification is not required when clinical improvement is clear [4]. Isolate-specific susceptibility testing was therefore deferred, and its absence did not appear to alter management or outcome.

Continued ophthalmologic examination confirmed a paracentral, shallower ring-shaped ulcer with preserved stromal integrity and no evidence of perforation. The peripheral cornea continued to clear. Pain was significantly reduced, though photophobia persisted. Visual acuity remained at counting fingers. The anterior chamber was clear without hypopyon; the pupil was dilated to 4 mm on cyclopentolate without synechiae; the lens was clear; funduscopy showed an attached retina with a bright red reflex.

Following microbiologic confirmation of *C. bovis *and sustained clinical improvement without evidence of progressive infection or impending perforation, the antimicrobial regimen was narrowed to reduce the cumulative ocular surface toxicity associated with prolonged, intensive fortified topical therapy while maintaining adequate coverage [4]. Vancomycin drops were reduced from four to three times daily; ceftazidime from four to three times daily; voriconazole from four to three times daily; neomycin/polymyxin B/gramicidin was reduced to twice daily; and povidone-iodine drops were reduced from four to two times daily. Tobramycin-dexamethasone ophthalmic solution was introduced at one drop to the left eye every 24 hours. The tobramycin component provided continued antibacterial coverage, while the low-dose corticosteroid was added judiciously to suppress residual stromal inflammation and limit corneal opacity formation, given that active infection was felt to be controlled [4].

Systemic piperacillin-tazobactam, intravenous vancomycin, and oral miltefosine were discontinued at discharge. The patient was discharged home on the adjusted topical ophthalmic regimen, which included voriconazole at the reduced frequency; voriconazole was discontinued when serial fungal cultures were finalized as negative at four weeks. Cyclopentolate 2% was continued for ongoing cycloplegia. Oral ciprofloxacin 500 mg twice daily for seven days and doxycycline 100 mg twice daily for seven days were prescribed at discharge. Ciprofloxacin served as empiric adjunctive systemic antibacterial therapy given the severity of keratitis and the risk of scleral extension or undetected polymicrobial infection [4]. Doxycycline was added not as primary antimicrobial coverage but for its role as a matrix metalloproteinase (MMP) inhibitor: by inhibiting corneal collagenase activity, it reduces the risk of progressive stromal degradation and perforation in active infectious keratitis, an adjunctive indication independent of its antibacterial properties [4,14]. The patient was instructed to follow up with Ophthalmology and Infectious Disease within three to five days of discharge and with his primary care physician for management of newly diagnosed type 2 diabetes mellitus.

**Figure 2:**
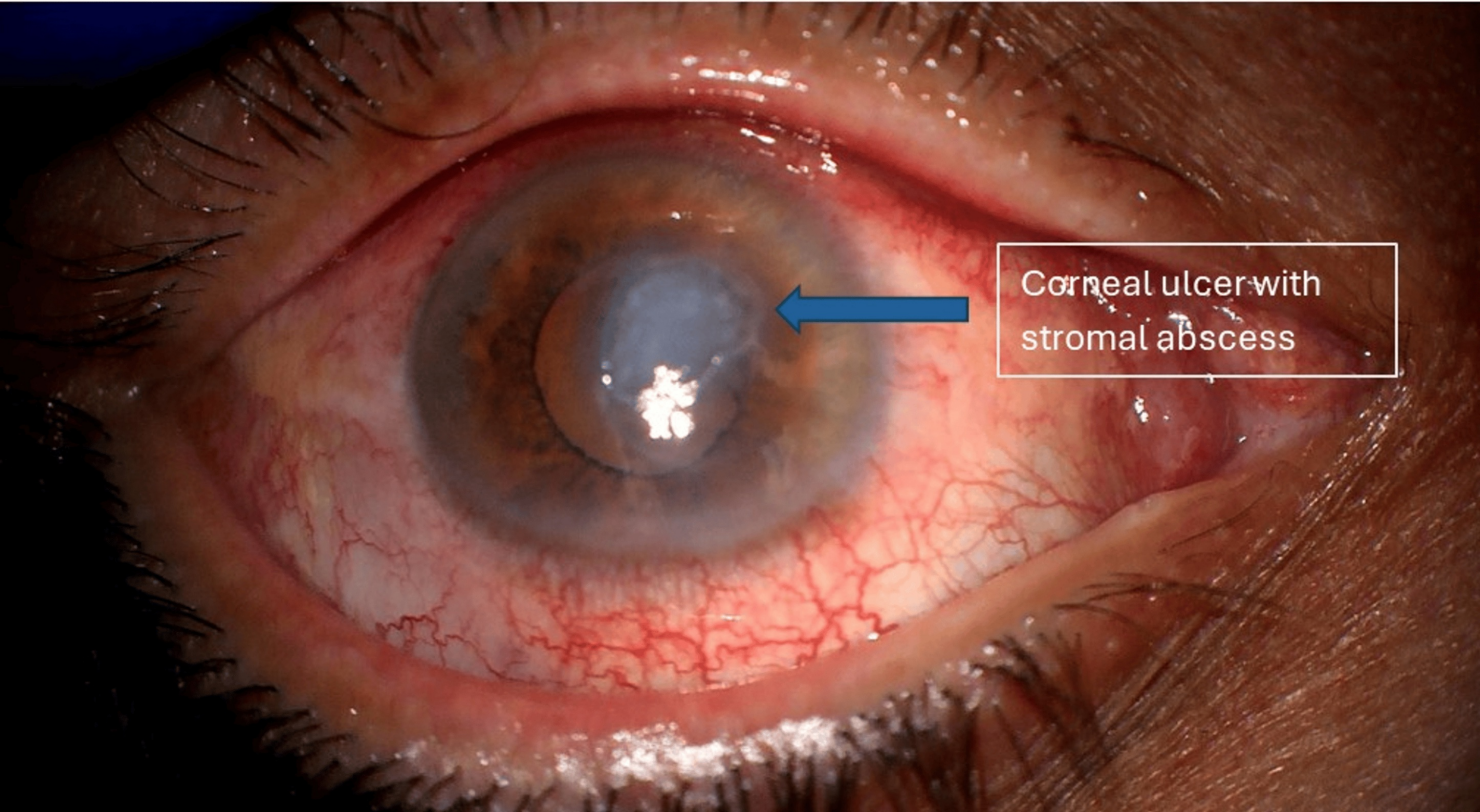
External photograph of the left eye at presentation for surgical management, showing a central corneal ulcer with an underlying stromal abscess (arrow) and diffuse conjunctival injection. The resolving hypopyon noted on slit-lamp examination is not appreciable on this diffuse-illumination external image.

Twenty-eight days after discharge, the patient returned for surgical management of a recurrent corneal ulcer with stromal abscess. Visual acuity at this visit was 20/40 in the right eye and 20/count fingers in the left eye. Slit-lamp examination revealed a central corneal ulcer with a stromal abscess (Figure 2) and a resolving hypopyon. The peripheral cornea was clear; there was no corneal perforation; and the anterior chamber was formed without endophthalmitis, cataract, or corneal scarring. He underwent surgical debridement of the left cornea with corneal epithelial scrapings and revision/repair of the anterior segment wound, including anterior chamber aqueous aspiration. Sub-Tenon's injections of ceftazidime (100 mg/0.5 mL), vancomycin (25 mg/0.5 mL), and triamcinolone (40 mg) were administered. Tobramycin/dexamethasone ointment was applied with a sterile patch. Cultures from both the corneal epithelial scrapings and the aqueous aspirate demonstrated no growth. Postoperatively, the patient received oral doxycycline 100 mg once daily for seven days; topical therapy included ceftazidime drops twice daily, moxifloxacin 0.5% drops three times daily, prednisolone acetate 1% drops four times daily, and neomycin-polymyxin B/dexamethasone ointment twice daily.

**Figure 3:**
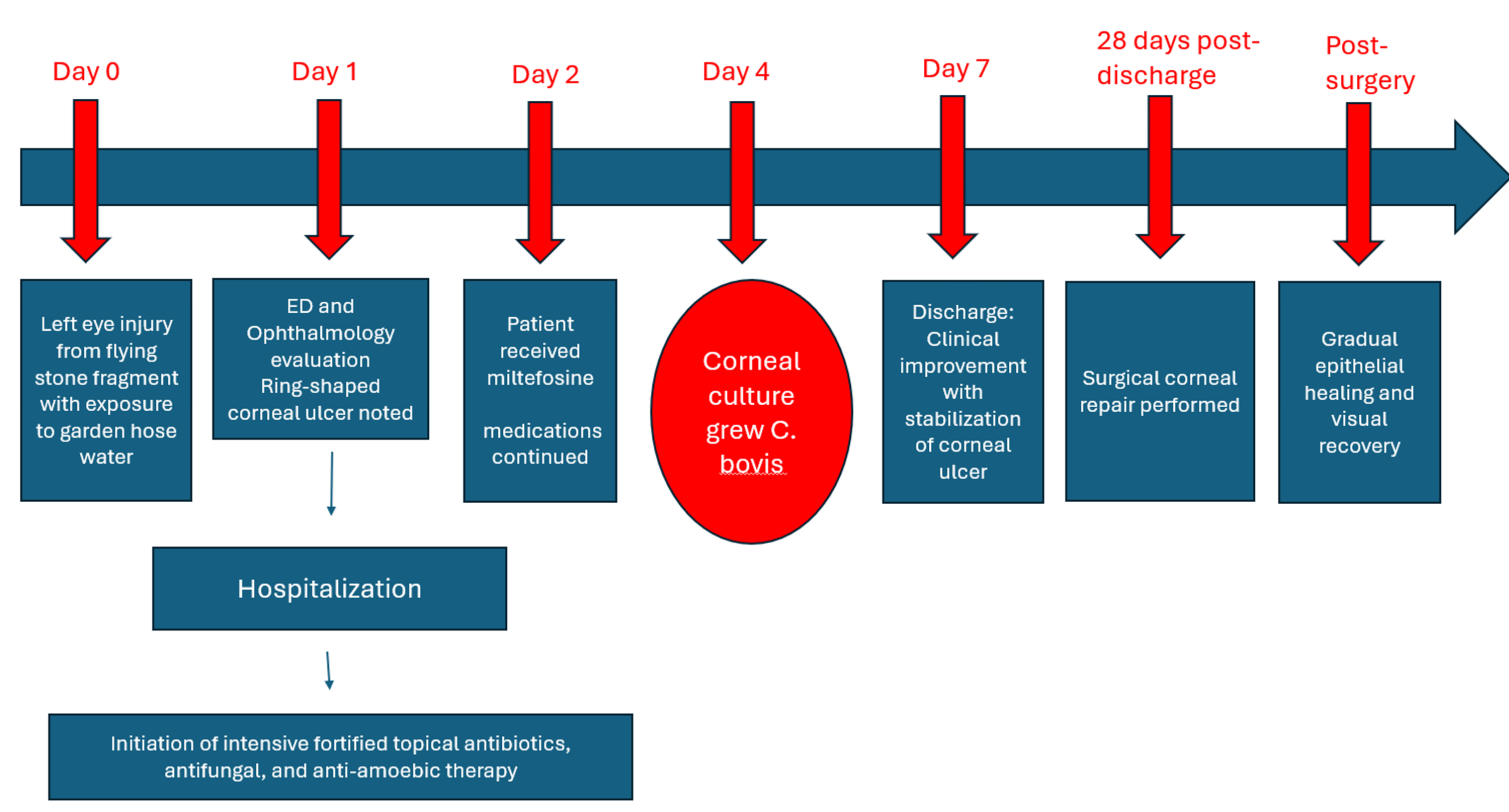
Timeline illustrating the patient’s clinical course from initial ocular injury to final recovery. The figure was manually created using Microsoft PowerPoint without the use of generative artificial intelligence.

Approximately four months after presentation, the patient underwent penetrating keratoplasty of the left eye, with the globe preserved and the graft in place at subsequent follow-up (Figure 3). Formal best-corrected visual acuity beyond the early postoperative period was not available.

## Discussion

*C. bovis* is an uncommon cause of infectious keratitis [1,2]. Although *Corynebacterium* species are frequently recovered from ocular cultures and often dismissed as contaminants, this case illustrates that they can act as true pathogens.

The ring-shaped stromal infiltrate, combined with contaminated water exposure and near-total visual loss at presentation, produced a clinical picture most closely resembling *Acanthamoeba* keratitis. A ring-shaped corneal infiltrate reflects a circular pattern of stromal inflammation, most often attributed to an immune-mediated host response or centrifugal organism spread within the corneal stroma; this pattern is most commonly associated with *Acanthamoeba *but may also occur in advanced fungal infection, making it an important but nonspecific finding [3,15]. Bacterial keratitis, *Acanthamoeba *keratitis, and fungal infection each remained viable diagnoses at presentation (Table 2) [3,8,16-19]. 

**Table 2:** Differential diagnosis of Acanthamoeba, bacterial, and fungal keratitis. The table was created based on established peer‑reviewed ophthalmology literature describing infectious keratitis and synthesized with clinical features relevant to the present case [4,6–10].

Feature	Bacterial keratitis	Acanthamoeba keratitis	Fungal keratitis
Demographics	Older patients (mean age ~54 years); equal gender distribution	Younger patients (≤39 years, 73%); female predominance (73%)	Male predominance (agricultural workers); adults aged 20-50 years
Geographic distribution	Worldwide; trauma-related cases in agricultural regions	Developed countries with high contact lens use	Highest in tropical/subtropical regions (Asia, Africa); 20-60% of culture-positive keratitis
Primary risk factors	Contact lens wear (35%); ocular surface disease (47%); trauma (44%); systemic immunosuppression (18%)	Contact lens wear (93%); water exposure to lenses (71%); swimming/showering with lenses	Ocular trauma with organic matter (most common, 91.9%); agricultural/outdoor work; previous corticosteroid use; contact lens wear
Symptom duration	Shorter duration; acute presentation; median 25 days to re-epithelialization	Prolonged (median 30 days from onset to diagnosis); median 113 days to re-epithelialization	Subacute presentation (5-10 days); median 30 days to re-epithelialization
Pain severity	Mild to severe pain	Severe, disproportionate to clinical findings (95%)	Moderate pain; eye pain followed by blurred vision
Stromal infiltrate pattern	Dense central infiltrate; fibrin in the anterior chamber; no ring pattern	Ring infiltrate (54%); radial keratoneuritis (46%); perineural infiltrates	Feathery/serrated margins (OR 3.92); raised/dry elevated slough; satellite lesions (OR 6.27); irregular infiltrate edge; immune ring
Anterior chamber	Hypopyon in severe cases; fibrin present (associated with bacterial)	Endothelial plaques (69%, OR 8.00); anterior uveitis (39%)	Hypopyon may be present; endothelial plaques (OR 8.00); immune ring; fibrin absent
Depth of lesion	Full-thickness stromal involvement	Variable; may be superficial (epithelial only)	Deeper stromal involvement (OR 2.97); entire cornea in chronic cases
Common pathogens	*Pseudomonas aeruginosa *(>40% in CL-related); *Staphylococcus *spp.; *Streptococcus pneumoniae *	*Acanthamoeba *species (trophozoites and cysts)	Filamentous fungi: *Fusarium*, *Aspergillus *(>95%); yeast: *Candida *spp. (temperate climates)
Clinical differentiation accuracy	66% accuracy in differentiation from fungal keratitis	Difficult to distinguish from fungal keratitis; high index of suspicion with ring infiltrate, endothelial plaque	66% accuracy in differentiation from bacterial; 89% probability if feathery margins + raised slough + no fibrin
Corneal perforation risk	Lower	Moderate	5-6 times more likely than bacterial keratitis
Treatment duration	Shorter; median 40 days until discontinuation of antimicrobials	Prolonged; median 100 days until discontinuation of antimicrobials	Intermediate; median 49 days until discontinuation of antimicrobials
Treatment regimen	Fluoroquinolone alone or vancomycin + tobramycin	Chlorhexidine + polyhexamethylene biguanide; systemic antibiotics if no response	Topical natamycin
Prognosis	Better outcomes than fungal or *Acanthamoeba *	Especially difficult to treat; often poor outcomes	Difficult to treat; poor outcomes; worse than bacterial

The depth of stromal involvement and concern for early abscess formation elevated the risk of corneal perforation, justifying immediate empiric therapy [5]. Vancomycin has demonstrated reliability in *Corynebacterium *keratitis, as most isolates are susceptible in vitro, while fluoroquinolone resistance is increasingly reported [5,6]. Elsheikh et al. described severe *C. bovis* keratitis complicated by abscess and perforation following cattle exposure, with clinical improvement observed only after topical vancomycin was added [6]. Early initiation of vancomycin and ceftazidime combination therapy in our patient was temporally associated with clinical stabilization without progression to perforation, as recommended for severe bacterial keratitis of undetermined etiology [7]; however, the simultaneous use of multiple antimicrobials precludes attribution of the response to any single agent.

The decision to treat empirically for *Acanthamoeba *rather than await culture confirmation is supported by the natural history of the disease: treatment delays consistently predict rapid stromal destruction and poor visual outcomes [18]. When standard PHMB and chlorhexidine preparations were unavailable, Boston Simplus® and povidone-iodine were used as bridge agents based on documented in vitro cysticidal activity [9,10]. This is not equivalent to standard anti-amoebic therapy, but the alternative, withholding treatment while a potentially sight-destroying infection progressed, was not clinically defensible [8,15,20].

Although *C. bovis* was isolated on Day 4 and fungal cultures were negative, voriconazole was not immediately discontinued. Fungal cultures require prolonged incubation and carry imperfect sensitivity, particularly when preceded by topical antimicrobial therapy [8,21]. The history of environmental trauma with soil and contaminated water, both risk factors for filamentous fungal keratitis, and the severity of stromal involvement justified a gradual, monitored approach to antifungal de-escalation rather than abrupt discontinuation. Antifungal therapy was tapered as clinical improvement continued and serial cultures were finalized as negative at four weeks.

Recovery of *C. bovis* in pure culture, confirmed by MALDI-TOF MS, together with compatible clinical features and sustained improvement on therapy, supports its role as the causative pathogen rather than an incidental diphtheroid contaminant such as *Corynebacterium xerosis *or *Corynebacterium amycolatum* [6,22]. Conversely, *C. bovis* has been recovered from a corneal ulcer as an incidental co-isolate while a different organism drove the infection [23], a reminder that culture positivity must be weighed against the clinical course rather than accepted or dismissed reflexively. Although susceptibility testing was not performed, it did not compromise management. The AAO Preferred Practice Pattern states that therapy modification is not required in the setting of clear clinical improvement [4]. Vancomycin was maintained as the primary agent given near-uniform susceptibility of *Corynebacterium *species documented across large in vitro series [5,13]; fortified topical antibiotics achieve corneal tissue concentrations substantially exceeding systemic minimum inhibitory concentrations [4], further supporting the adequacy of the empiric regimen.

Unlike the previously reported case of *C. bovis* keratitis associated with zoonotic exposure and corneal perforation [6], this case suggests trauma-related environmental inoculation. Corneal epithelial disruption likely permitted organism entry, while uncontrolled diabetes (HbA1c 12.1%) probably contributed to impaired corneal repair and the protracted clinical course. Chronic hyperglycemia disrupts corneal epithelial function through impaired epidermal growth factor receptor (EGFR) signaling, a pathway essential for epithelial repair; reduced EGFR activity limits epithelial cell survival and renewal, contributing to delayed healing and epithelial instability [24]. Poor glycemic control was a likely contributor to the severity and duration of this infection, and its management should be considered part of the treatment plan rather than an ancillary concern.

This case expands the limited literature on *C. bovis* keratitis by demonstrating that severe infection may clinically mimic *Acanthamoeba *keratitis and carry a risk of corneal perforation requiring surgical intervention. In severe keratitis, maintaining a broad differential and interpreting diphtheroid isolates within the full clinical context are important; dismissing them as incidental contaminants without corroborating evidence risks a missed or delayed diagnosis.

Several limitations of this report should be noted. As a single-patient case, it is descriptive and cannot establish causality or support generalization. The patient received early, aggressive, multiple simultaneous therapies, making it difficult to attribute improvement to any single agent. Management included off-label and nonstandard bridge therapies (miltefosine and antiseptic agents with anti-amoebic activity) that may not be accessible in all clinical settings. Microbiologic evaluation relied on culture-based methods, and a polymicrobial process cannot be entirely excluded. Formal antimicrobial susceptibility testing was not performed on the isolate. Long-term visual outcomes were not assessed, as follow-up was limited to the early treatment course. These constraints are characteristic of managing a rapidly progressive infection in which empiric treatment must often precede complete microbiologic characterization.

## Conclusions

*C. bovis* should be recognized as a rare but clinically significant cause of infectious keratitis, and diphtheroid corneal isolates warrant clinical correlation rather than automatic dismissal. This case demonstrates that *C. bovis *keratitis can mimic *Acanthamoeba *infection with a ring-shaped stromal infiltrate, requiring broad empiric coverage until culture results are available. Early culture-directed therapy with prompt microbiologic sampling likely contributed to globe preservation, although surgical intervention was ultimately required.
